# Targeted Genomic Screen Reveals Focal Long Non-Coding RNA Copy Number Alterations in Cancer Cell Lines

**DOI:** 10.3390/ncrna4030021

**Published:** 2018-09-13

**Authors:** Pieter-Jan Volders, Steve Lefever, Shalina Baute, Justine Nuytens, Katrien Vanderheyden, Björn Menten, Pieter Mestdagh, Jo Vandesompele

**Affiliations:** 1 Center for Medical Genetics (CMGG), Ghent University, 9000 Ghent, Belgium; Steve.Lefever@UGent.be (S.L.); shalina.baute@ugent.be (S.B.); Justine.Nuytens@UGent.be (J.N.); Katrien.Vanderheyden@UGent.be (K.V.); Bjorn.Menten@UGent.be (B.M.); Pieter.Mestdagh@UGent.be (P.M.); Jo.Vandesompele@UGent.be (J.V.); 2 Cancer Research Institute Ghent (CRIG), 9000 Ghent, Belgium; 3 Bioinformatics Institute Ghent N2N (BIG N2N), 9000 Ghent, Belgium

**Keywords:** lncRNA, long non-coding RNA, array CGH, somatic copy-number alterations

## Abstract

The landscape of somatic copy-number alterations (SCNAs) affecting long non-coding RNAs (lncRNAs) in human cancers remains largely unexplored. While the majority of lncRNAs remain to be functionally characterized, several have been implicated in cancer development and metastasis. Considering the plethora of lncRNAs genes that have been currently reported, it is conceivable that many more lncRNAs might function as oncogenes or tumor suppressor genes. We devised a strategy to detect focal lncRNA SCNAs using a custom DNA microarray platform probing 10,519 lncRNA genes. By screening a panel of 80 cancer cell lines, we detected numerous focal aberrations targeting one or multiple lncRNAs without affecting neighboring protein-coding genes. These focal aberrations are highly suggestive for a tumor suppressive or oncogenic role of the targeted lncRNA gene. Although functional validation remains an essential step in the further characterization of the involved candidate cancer lncRNAs, our results provide a direct way of prioritizing candidate lncRNAs that are involved in cancer pathogenesis.

## 1. Introduction

The cancer genome is marked by large numbers of genetic and non-genetic alterations. The greater majority of those are somatic. Only a small fraction of the somatic mutations, the so-called driver mutations, contribute to cancer development by activating or inactivating specific cancer genes. The remainder are passenger mutations that do not confer a growth advantage, but were acquired at some point during cancer cell proliferation [1]. Differentiating between driver and passenger mutations is one of the biggest challenges in the quest for new cancer genes and putative therapeutic targets. While somatic alterations can be as small as a single nucleotide substitution, insertion or deletion, somatic copy-number alterations (SCNAs) affect the largest fraction of the genome [2]. In some cases, SCNAs affect entire or partial chromosome arms. The ability to detect these genetic/genomic alterations using (molecular) cytogenetic methods has made large SCNAs historically the best studied cancer-associated genetic alterations. Many well-known oncogenes and tumor suppressor genes have been initially identified as targets of recurrent genomic amplifications or deletions, respectively. Notable examples are tumor suppressor genes *PTEN* [3] and *RB1* [4], and oncogenes *HER2* (*ERBB2*) [5] and the *MYC*-family of transcription factors [6,7]. The resulting diagnostic and therapeutic successes have made cancer SCNAs the subject of many studies. Additionally, the advent of array comparative genome hybridization (array CGH) platforms that enable the robust identification of small SCNAs have greatly improved our knowledge of the cancer genome [8,9,10].

As cancer genetics until now mainly focused on protein-coding genes, not much is known about SCNAs that affect non-coding RNA genes in cancer. In recent years, our knowledge on the non-coding genome has expanded enormously. This is especially the case for the class of long non-coding RNAs (lncRNAs), consisting of genes with transcripts that are larger than 200 nucleotides that do not encode proteins. In the past five years, tens of thousands of human lncRNAs have been reported and catalogued, making this the largest genetic class in the human genome [11]. While the bulk of lncRNAs remains to be functionally annotated, they have been implicated in many important normal cellular processes such as dosage compensation [12], chromatin remodeling [13], and cell differentiation [14]; when deregulated, they play a role in disease as well, including cancer [15].

The discovery of cancer-associated lncRNAs such as *HOTAIR* [16], *MALAT1* [17], and *PVT1* [18] uncovered an important role for lncRNAs in oncogenesis. The reason for the current hiatus in our knowledge on lncRNA SCNAs is the fact that the majority of lncRNA annotations are very recent. Most commercially available platforms or reference databases are based on older genomic annotations (with no probes for lncRNAs, or probes for as-yet unannotated lncRNAs), or lncRNAs are simply overlooked in the data analysis. Indeed, recurrent SCNAs outside of protein-coding regions have been reported [2,19]. To overcome this problem, existing DNA microarray platforms have been repurposed, and probe content has been reannotated with current lncRNA annotation [20,21]. One such effort resulted in the discovery of the oncogenic focally amplified lncRNA on chromosome 1 (*FAL1*) [21,22] and the ovarian adenocarcinoma amplified lncRNA (*OVAAL*) [23] lncRNAs, which are implicated in epithelial and ovarian cancers. While the potential of this approach lies in its ability to make use of the large amount of publically available DNA microarray data, the platforms used have several disadvantages for the discovery of putative cancer-associated lncRNAs. Whole cancer genome sequencing has the potential in principle to circumvent these limitations, but the method is still relatively expensive and challenging in terms of data analysis. Consequently, public databases (e.g., The Cancer Genome Atlas (TCGA, National Cancer Institute, Bethesda, MD, USA)) are mainly populated with targeted exome sequencing datasets, again focusing on protein-coding genes. Lastly, while shallow whole cancer genome sequencing is a cost-effective method to detect copy number variations, its coverage is limited to large events.

Here we present a targeted and cost-effective approach to identify focal lncRNA SCNAs that is based on a custom DNA microarray covering 10,519 lncRNA genes and their flanking protein-coding genes. We show that this platform has the ability to detect focal aberrations that only affect lncRNA exons, and it does not encompass their flanking protein-coding genes. By analyzing the DNA of 80 cell lines from 11 cancer types, we reveal that lncRNAs are frequently targeted by focal aberrations in human cancer. In addition, we have generated a dataset with putative oncogenic and tumor suppressor lncRNAs for future functional studies.

## 2. Results

### 2.1. A Targeted Platform to Detect Focal Copy Number Changes in lncRNA Genes

Long non-coding RNAs are underrepresented on commercial array CGH platforms, and the mean chromosomal distance between the probes on these arrays makes them unsuitable for detecting small aberrations that only involve (part of) a single lncRNA gene (Figure S1).

In order to detect small and focal SCNAs that only affect lncRNA exons, we designed a custom 180K CGH array covering intergenic lncRNA exons, and the nearest exons of their flanking protein-coding genes. To this purpose, we constructed a database with 52,324 non-redundant exons derived from all transcripts listed in LNCipedia 1.0 (Figures S2 and S3) [11]. The database was subsequently extended with protein-coding gene annotation from Ensembl (EMBL-EBI, Wellcome Genome Campus, Hinxton, UK). Next, we designed probes using the genomic sequence of the lncRNA exons, and the two nearest exons of the flanking protein-coding genes. By removing duplicate probes in overlapping exons and selecting additional probes for transcripts with fewer exons, we were able to cover the majority (94%) of the transcripts with at least 10 probes (Figure S4). Only 1.2% of lncRNAs could not be covered by any probe. For 95% of the lncRNA transcripts, we succeeded in designing two probes for each flanking protein-coding exon.

To assess the quality of our custom array CGH platform, we compared the profiles for 60 cancer cell lines (NCI-60 subset) to publically available profiles of two different array CGH platforms. The average log ratio in 1 Mb bins was calculated and correlated between the different platforms. These correlations were compared with correlations among unrelated cell lines (Figures S5 and S6). Correlation between the same cell lines across the different platforms was high (median Pearson’s correlation = 0.70), validating the quality of our profiles. As expected, the cell lines derived from the same individual (such as National Cancer Institute (NCI)/ADR-RES and OVCAR-8) were also highly correlated (Pearson’s correlation = 0.74). In addition, this analysis revealed problems with two DNA samples (HCT-15 and CAKI-1), as the obtained profiles showed poor correlations with publically available profiles. This poor correlation remained unresolved after repeating the hybridization. As such, results from these two cell lines should be interpreted with care.

### 2.2. Frequent Focal lncRNA Copy Number Alterations in Cancer Cell Lines

**Figure 1:**
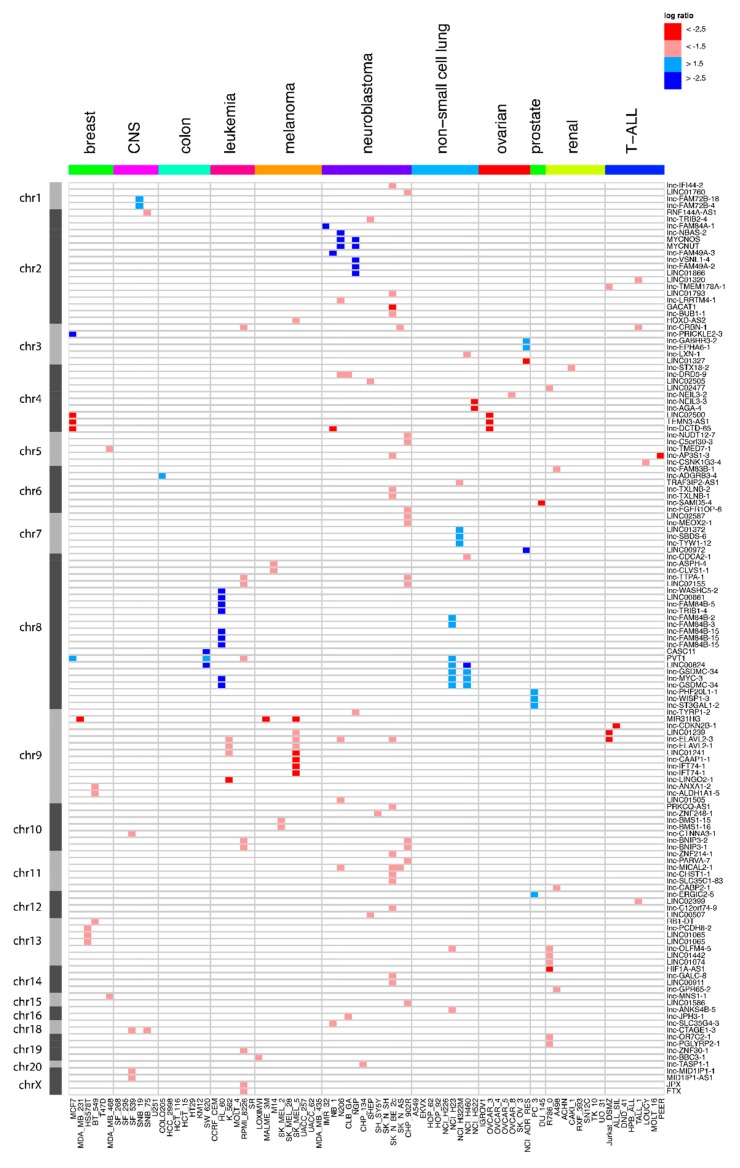
Overview of the long non-coding RNA (lncRNA) genes affected by focal somatic copy-number alterations (SCNAs) after extensive filtering. Red represents the copy number loss (log-ratio < 1.5) in that cell line, while blue corresponds to copy number gain (log-ratio > 1.5). Dark red and blue correspond to copy number changes with absolute log-ratios of above 2.5.

To explore focal lncRNA SCNAs in cancer, we analyzed DNA from 80 cancer cell lines covering 11 cancer types with our custom DNA microarray (Table 1). An extensive filter was performed on the resulting segments to shortlist focal lncRNA SCNA alterations. To be considered a lncRNA SCNA, a segment should (1) overlap with the exonic lncRNA sequence; (2) not be contained within known segmental duplications; (3) overlap with at most three known variants, and (4) have an absolute average log-ratio that is larger than 1.5 (reflecting homozygous deletions and gene amplifications). In the case of an amplification, an additional requirement was that the segment included the entire transcript. Finally, to withhold a focal SCNA (5), the segment cannot overlap any of the flanking protein-coding gene exons. Using these settings, 173 focal SCNAs affecting 136 lncRNAs in at least one cell line were identified (Figure 1, Table S1). The majority of these lncRNAs (111) were affected in a single cell line, 16 are affected in two cell lines, 7 in three cell lines, one in four cell lines, and one in five cell lines. By confining the relative difference in log-ratio between the segment covering the lncRNA and the segment covering the flanking protein-coding genes, it was possible to retain the superimposed SCNAs (for instance, a large hemizygous deletion that contains a smaller homozygous deletion). A more stringent subset of 76 lncRNA SCNAs was obtained if we required that the flanking protein-coding gene did not show any copy number change (Figure S7, Table S2). This stringent set excluded stacked events, e.g., a homozygous deletion in a larger heterozygous deletion that encompasses one of the flanking protein-coding genes.

### 2.3. Quantitative Polymerase Chain Reaction Confirms the Majority of Focal Aberrations

**Figure 2:**
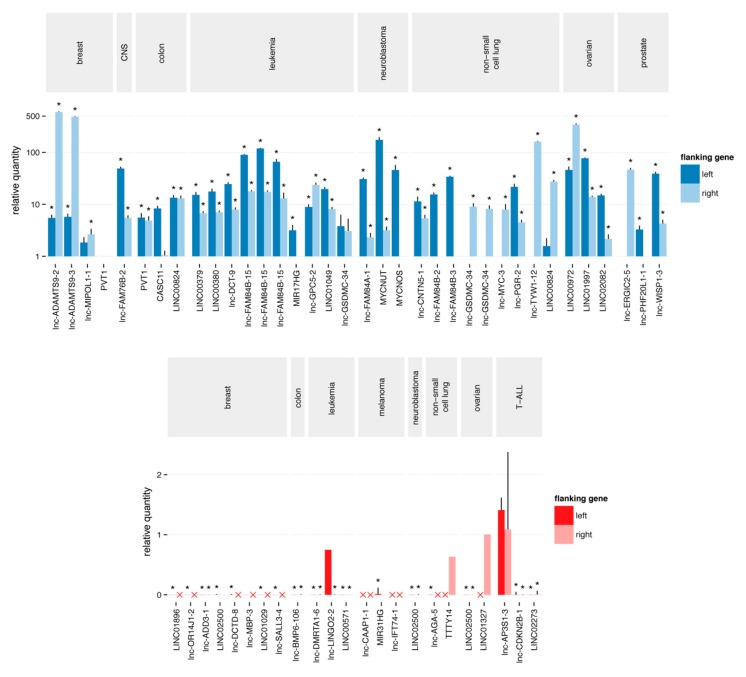
Quantitative polymerase chain reaction validation of the putative focal SCNAs. The Cq value of the aberration is normalized to the Cq value of each of the flanking regions. A copy number gain (blue) is considered as confirmed and focal when the relative quantity to both flanking regions is higher than one. Similarly, a copy number loss (red) is considered as confirmed and focal when the relative quantity to both flanking regions is less than one. Red crosses represent Cq values > 35, corresponding to a homozygous deletion of the flanking regions. Stars represent significant (*p*-value < 0.05) differences from one.

We devised a unique strategy to validate the selected focal lncRNA SCNAs using quantitative polymerase chain reaction (qPCR). Assays were designed by targeting the genomic locus of the aberration and the nearest exons of the flanking protein-coding genes. By comparing the Cq value of the lncRNA locus and the flanking coding exons, we could accurately assess the difference in copy number between the two. Using this strategy, we evaluated 88 events (Figure 2). For 66 of these (75%), an altered copy number status compared to at least one of the two flanking assays could be confirmed, of which 43 (49%) showed an expected relative difference in Cq values with both flanking assays, and they were thus validated as focal aberrations. The validation rate was higher for the amplifications than for the deletions (56% and 48%, respectively). The validation rate drastically increased when we limited our analysis to the subset of segments with an absolute average log-ratio that was larger than 2.5. In that case, 58 out of 64 (91%) events were confirmed to be copy number alterations. The fraction of confirmed focal aberrations remained similar (53%).

### 2.4. Most Novel lncRNA Aberrations do not Correspond to Common Somatic Variants

As our custom platform differed considerably from other array CGH platforms, it was not unlikely that the newly found SCNAs actually comprised uncharted germline copy-number variants that may exist in a normal population and that do not contribute to cancer. To assess this possibility, we performed an qPCR experiment for five validated loci on DNA from 192 healthy individuals. Neither homozygous deletions nor high order amplifications could be detected for any lncRNAs in any of the samples (Figure S8). Of note: for one lncRNA, heterozygous deletions were found in 12 individuals (6%).

## 3. Discussion

Even though the number of samples that we examined was limited and confined to cell lines, we were able to detect a large number of SCNAs that specifically affect lncRNA exons. This suggests that similarly to protein-coding genes, lncRNAs are frequently targeted by SCNAs in cancer. After rigorous filtering, focusing on novel highly aberrant segments that not encompass protein-coding genes, we report 136 such events, including 25 that are recurrent. In 76 of the 136 events, the flanking protein-coding genes are copy number neutral. Since the cancer genome harbors many large SCNAs, it is important to also consider the events where the flanking protein-coding genes are not strictly copy number normal, as long as the lncRNA itself is focally affected by a second event as well. 

**Figure 3:**
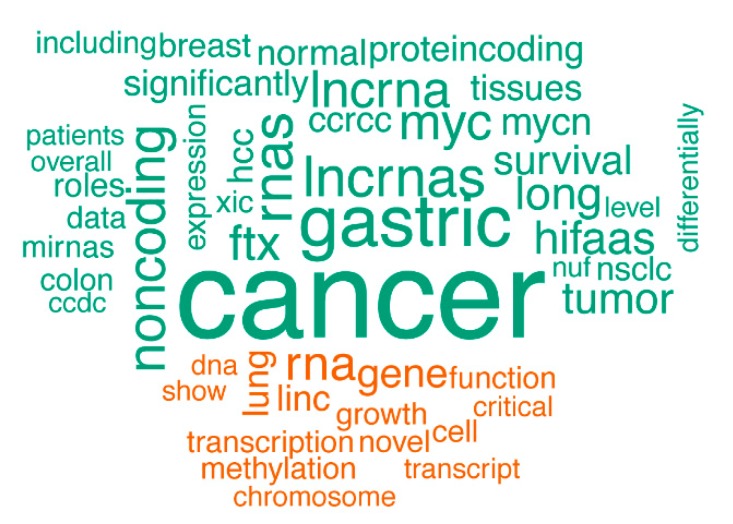
A comparison word cloud shows strong enrichment of the word “cancer” in abstracts of publications associated with the lncRNAs affected by SCNAs. The size of each word corresponds to the deviation of its frequency in abstracts associated with the lncRNAs affected by SCNAs from the average occurrence frequency. Green words are more prevalent in abstracts on lncRNAs affected by SCNAs, while orange words are more prevalent in the abstracts on lncRNAs unaffected by SCNAs.

Our strategy uncovered several cancer-associated lncRNAs. For instance, the known oncogene *PVT1* was detected as a recurrent focal aberration (Figure 1 and Figure S3). *PVT1* has been implicated in several cancer types including gastric cancer [24], ovarian cancer and breast cancer [18]. *PVT1* was found to be co-amplified in more than 98% of cancers with a *MYC* copy number increase [25]. Our work not only confirms the frequent amplification of *PVT1* in cancer, but it also reveals that *PVT1* amplifications can be focal. Another interesting concurrence with previous studies is found in a large-scale pan-cancer study on SCNAs [19]. Although the authors mainly focus on SCNAs that affect protein-coding genes and use limited lncRNA annotation, they report one lncRNA, LINC00290, as the sole member of a frequently deleted region. Our results reveal a recurrent and focal deletion in ovarian and breast cancer cell lines, suggesting a role in multiple cancer types (Figure 1). Recently, Lanzós and colleagues identified 15 cancer-driving lncRNAs based on somatic single nucleotide variants (SNVs) in tumor samples [26]. Our study finds one of those candidates, LINC01505, in a focal deletion in a neuroblastoma cell line. Furthermore, text mining of the abstracts of publications associated with the lncRNAs affected by SCNAs in our screen showed a clear enrichment of the word “cancer” (*p* = 3.608 × 10^−14^) (Figure 3). This analysis further underscores the potential of our approach to enrich for cancer-related lncRNAs.

The validation rate determined by qPCR was strongly dependent on the log-ratio cutoff applied to the segments, with an absolute average log-ratio larger than 2.5 showing high validation rates for the lncRNA copy number status. The relatively high cutoff is likely to be related to the unique design of our platform. As the probes are confined to small genomic loci (lncRNA exons) it is not unimaginable that the observed signal-to-noise ratio is different compared to the typical designs. In addition, qPCR may not be the most appropriate method to detect hemizygous copy number changes. Even with a stringent log-ratio cutoff (2.5), only 50% of the events could be confirmed to be truly focal. This suggests that the limited number of probes on the flanking protein-coding genes is insufficient to define the breakpoints of the segments in some cases.

Nevertheless, even when taking the validation rate into account, our research finds about 100 lncRNAs affected by focal SCNA. As the majority of these events are likely, no germline copy-number variants, these SCNAs harbor interesting candidates for further research.

## 4. Materials and Methods 

### 4.1. A lncRNA Exon Database

Annotation of lncRNA transcripts was obtained from LNCipedia [27] (version 1.0) and stored in a MongoDB NoSQL database (MongoDB, Inc., New York, NY, USA). Protein-coding transcript annotation was obtained from Ensembl’s [28] biomart (version 64, September 2011) and stored in the same format. For every lncRNA transcript, the nearest upstream and downstream protein-coding transcripts were determined. To interface with the MongoDB dataset, both perl scripts and mongo shell scripts were employed. Using MongoDB’s MapReduce functionality, a non-redundant exon collection was built starting from the collection of non-redundant transcripts. 

All lncRNA identifiers used in this work, such as in the tables and captions are based on LNCipedia 5.2.

### 4.2. Array Comparative Genome Hybridization Platform Design

An array CGH probe design was performed using Agilent Technologies eArray software (https://earray.chem.agilent.com/earray/). A Browser Extensible Data (BED) file of all non-redundant exons was generated from the exon database and uploaded into eArray for probe design. Since our criterion to have two probes per exon was initially not met, the exon boundaries were extended and the corresponding BED files were uploaded as well. The exon boundaries were extended by 100 bp, 300 bp, and 500 bp. In addition, less stringent selection parameters were used for the 500 bp-extended exon. In this way, five probe datasets were generated and stored in a separate MongoDB collection. From this collection, two probes per exon (neighborhood) were selected with preference for the probes closest to the exon. Overlapping transcripts were taken into account, to avoid duplicate probe selection. For transcripts with fewer than five exons, additional probes were selected until the transcript was covered by at least 10 probes. For the flanking protein-coding genes, probes were designed for the two exons closest to the lncRNA. From this set, the two probes nearest to the lncRNA were selected. The resulting set of 166,417 unique probes was uploaded to eArray and supplemented with normalization and quality control probe groups recommended by Agilent Technologies. Agilent Technologies subsequently manufactured the final design in the 4 × 180 K format. The design of the platform was made publically available through the Gene Expression Omnibus (GEO) website using the accession number GPL22307.

### 4.3. Cancer Cell Line DNA and RNA

The NCI provided DNA and RNA samples for all cell lines in the NCI-60 cancer cell line panel. Neuroblastoma and T-ALL cell lines were available in house; RNA extraction was performed with the miRNeasy Mini Kit (Qiagen N.V., Venlo, the Netherlands), and DNA extraction with the QIAamp DNA Mini Kit (Qiagen).

### 4.4. Array Comparative Genome Hybridization

Four hundred nanograms of genomic DNA was labeled with Cy3-dCTP (GE Healthcare, Machelen, Belgium) using a Bioprime array CGH genomic labeling system (Thermo Fisher Scientific, Waltham, MA, USA). In parallel, Kreatech gender-matched controls were labeled with Cy5-dCTP. Samples were hybridized on the custom array CGH arrays for 40 h at 65 °C. After washing, the samples were scanned at 5 µm resolution using a DNA microarray scanner, G2505B (Agilent Technologies, Santa Clara, CA, USA). The scan images were analyzed using the feature extraction software 9.5.3.1 (Agilent Technologies). Segmentation was achieved using the circular binary segmentation algorithm in the DNACopy R package. Visual inspection and creation of the copy number profile plots were performed with ‘Vivar’ [29]. All of the raw array CGH data files were made publically available through the GEO website using the accession number GSE85444.

### 4.5. Segment Analysis and Filtering

Segment position and statistics are stored in a MongoDB collection. A perl script was used to combine the segment annotation with lncRNA and protein-coding gene annotations in other collections, and to implement the filtering process. First, only segments that overlapped the lncRNA exons were retained. Next, segments with an absolute average log-ratio of less than 1.5 were discarded, as were segments contained within segmental duplications (UCSC genomicSuperDups track), or segments that overlapped with more than three known variants (database of genomic variants [30]). The absolute log-ratio of the nearest segments covering the flanking protein-coding genes should be 0.5 lower than the segment covering the lncRNA (corresponding to about one copy less). A more stringent subset of segments was obtained by requiring the absolute log-ratio of the nearest segments covering the flanking protein-coding genes to be less than 0.35 (copy number neutral).

### 4.6. Quantitive PCR Validation

Quantitative PCR assays were designed, based on the chromosomal locations of the altered segment covering the lncRNA and the nearest exons of the two flanking protein-coding genes. Primer design was performed using Primer3 [31]; primers spanning common single nucleotide polymorphisms (SNPs) were excluded. The specificity is evaluated using BiSearch [32]. All qPCR reactions were prepared using Bio-Rad’s SsoAdvanced Universal SYBR Green Supermix in a 5 µL volume (2.5 µL mastermix, 0.25 µL each of forward and reverse primers (250 nM final concentration) and 2 µL DNA (5 ng)). Quantitative PCR plates were run on the LightCycler 480 (Roche Life Science, Indianapolis, IN, USA) using 2 min activation at 95 °C, followed by 45 cycles of 5 s at 95 °C, 30 s at 60 °C, and 1 s at 72 °C, and a melt curve analysis.

The calculation of normalized relative quantities was done using qbase+ software version 2.6 (Biogazelle NV, Ghent, Belgium) and the open source statistical environment R (version 3). The Cq values corresponding to the altered segment were normalized to those corresponding to the flanking protein-coding genes, and scaled to the control sample (Human Genomic DNA, Roche Life Science, Indianapolis, IN, USA). Downstream analysis and data visualization was achieved using R and third party modules (plyr, ggplot2).

### 4.7. Text Mining

The abstract texts of 2384 curated articles on lncRNAs were obtained from LNCipedia 5.1. These were subdivided into 127 abstracts on lncRNAs affected by a SCNA in our data and 2257 abstracts on lncRNAs not affected by SCNAs. Next, the abstract text was analyzed in R (version 3.4.4) using the tidyverse (version 1.2.1), stringi (version 1.2.2), word cloud (version 2.5), and tm (version 0.7-3) packages. In brief: punctuation, stopwords, lncRNA names, and numbers were removed, and the resulting texts were transformed to a term-document matrix that stored the frequency of all words in the different groups. The matrix was subsequently used to create a comparison word cloud. The statistical significance of the enrichment of selected words in the abstracts associated with lncRNAs affected by a SCNA was tested using Fisher’s exact test. 

## 5. Conclusions

We developed and applied a unique array CGH platform that was capable of detecting small and focal lncRNA SCNAs. We have screened a panel of 80 cancer cell lines and shortlisted 136 lncRNA genes with a putative role in cancer. Among this list are several lncRNAs that have been implicated in cancer, validating our approach. Since the great majority of the lncRNAs on our platform have yet to be functionally studied, this finding suggests that our research provides many new cancer-related lncRNA genes. We present a set of lncRNA genes to the lncRNA and cancer research community as novel candidate cancer lncRNA genes for further functional exploration.

## Figures and Tables

**Table 1:** Overview of the cell line panel and the cell line origins.

Cancer Type	#	Cell Lines	Origin
Breast	6	MCF7, MDA-MB-231, HS578T, BT-549, T47D, MDA-MB-468	NCI
CNS	6	SF-268, SF-295, SF-539, SNB-19, SNB-75, U251	NCI
Colon	7	COLO205, HCC-2998, HCT-116, HCT-15, HT29, KM12, SW-620	NCI
Leukemia	6	CCRF-CEM *, HL-60, K-562, MOLT-4 *, RPMI-8226, SR	NCI
Melanoma	9	LOXIMVI, MALME-3M, M14, SK-MEL-2, SK-MEL-28, SK-MEL-5, UACC-257, UACC-62, MDA-MB-435	NCI
Non-small cell lung	9	A549, EKVX, HOP-62, HOP-92, NCI-H226, NCI-H23, NCI-H322M, NCI-H460, NCI-H522	NCI
Ovarian	7	IGROV1, OVCAR-3, OVCAR-4, OVCAR-5, OVCAR-8, SK-OV-3, NCI-ADR-RES	NCI
Prostate	2	PC-3, DU-145	NCI
Renal	8	786-0, A498, ACHN, CAKI-1, RXF-393, SN12C, TK-10, UO-31	NCI
T-cell acute lymphoblastic leukemia	8	Jurkat-DSMZ, ALL-SIL, DND-41, HPB-ALL, TALL-1, LOUCY, MOLT-16, PEER	DSMZ
Neuroblastoma	12	CLB-GA, IMR-32, NB-1, NGP, N206, SHEP, SH-SY5Y, SK-N-SH, SK-N-BE-2c, CHP-134, SK-N-AS, CHP-902R	CMGG
11 types	80		

* MOLT-4 and CCRF-CEM are T-cell acute lymphoblastic leukemia cell lines in the NCI60 panel. CNS = central nervous system, NCI = National Cancer Institute.

## Supplementary Materials

The following are available online at http://www.mdpi.com/2311-553X/4/3/21/s1, Figure S1: The smallest theoretical segment that covers each lncRNA using the Genome-Wide Human SNP Array 6.0; Figure S2: Distribution of the number of exons for the lncRNA transcripts in our dataset; Figure S3: Distribution of the number of transcripts per lncRNA gene (locus); Figure S4: The required number of probes for a transcript depends on the number of exons; Figure S5: Comparison of the global copy number profiles (averaged in 1 Mb bins) with the publically available profiles of two different array CGH platforms (Agilent 44K and Nimblegen 385k); Figure S6: Complete linkage tree of the cell line based on their global copy number profiles; Figure S7: Overview of the lncRNA genes affected by focal SCNAs and the copy number normal flanking protein-coding genes; Figure S8: qPCR validation of five selected loci on 192 DNA samples from healthy individuals; Table S1: Overview of the lncRNA genes affected by focal SCNAs after extensive filtering and their copy number status in the tested cell lines; Table S2: Overview of the lncRNA genes affected by focal SCNAs and the copy number of the normal flanking protein-coding genes, and their copy number status in the tested cell lines.
