# Supplementary material for: Targeted Genomic Screen Reveals Focal Long Non-Coding RNA Copy Number Alterations in Cancer Cell Lines
Source: Noncoding RNA. 2018 Sep 13;4(3):21. doi: 10.3390/ncrna4030021 (PMC6162788; doi:10.3390/ncrna4030021)
Supplement: Supplementary file 1 [file ncrna-04-00021-s001.zip › ncrna-351341-SI/ncrna-351341-finalsupplementary/Figures S1-S8.docx]

**
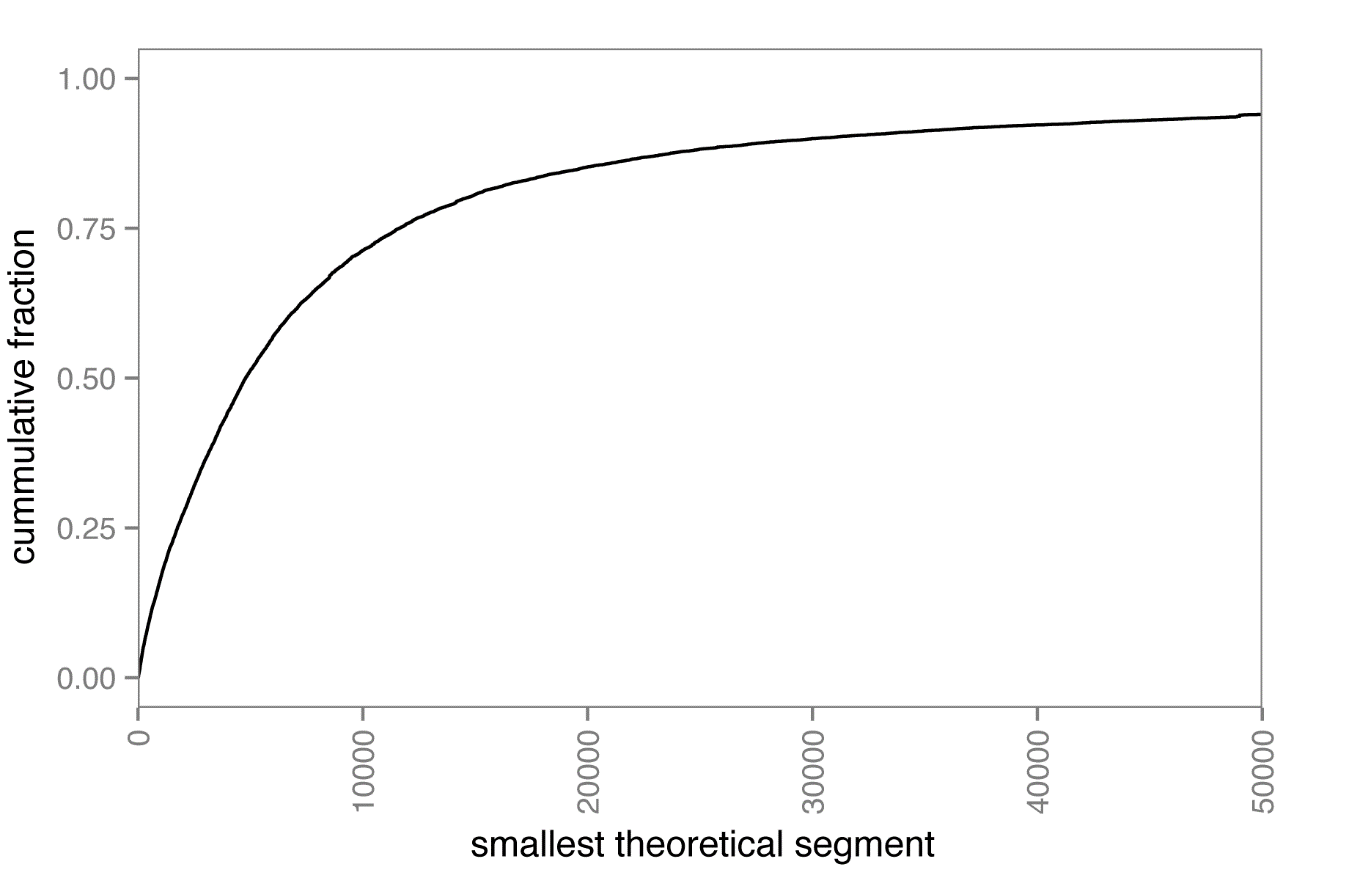
Figure S1:** The smallest theoretical segment that covers each lncRNA using the Genome-Wide Human SNP Array 6.0. A theoretical segment is the distance between the two closest probes covering lncRNAs


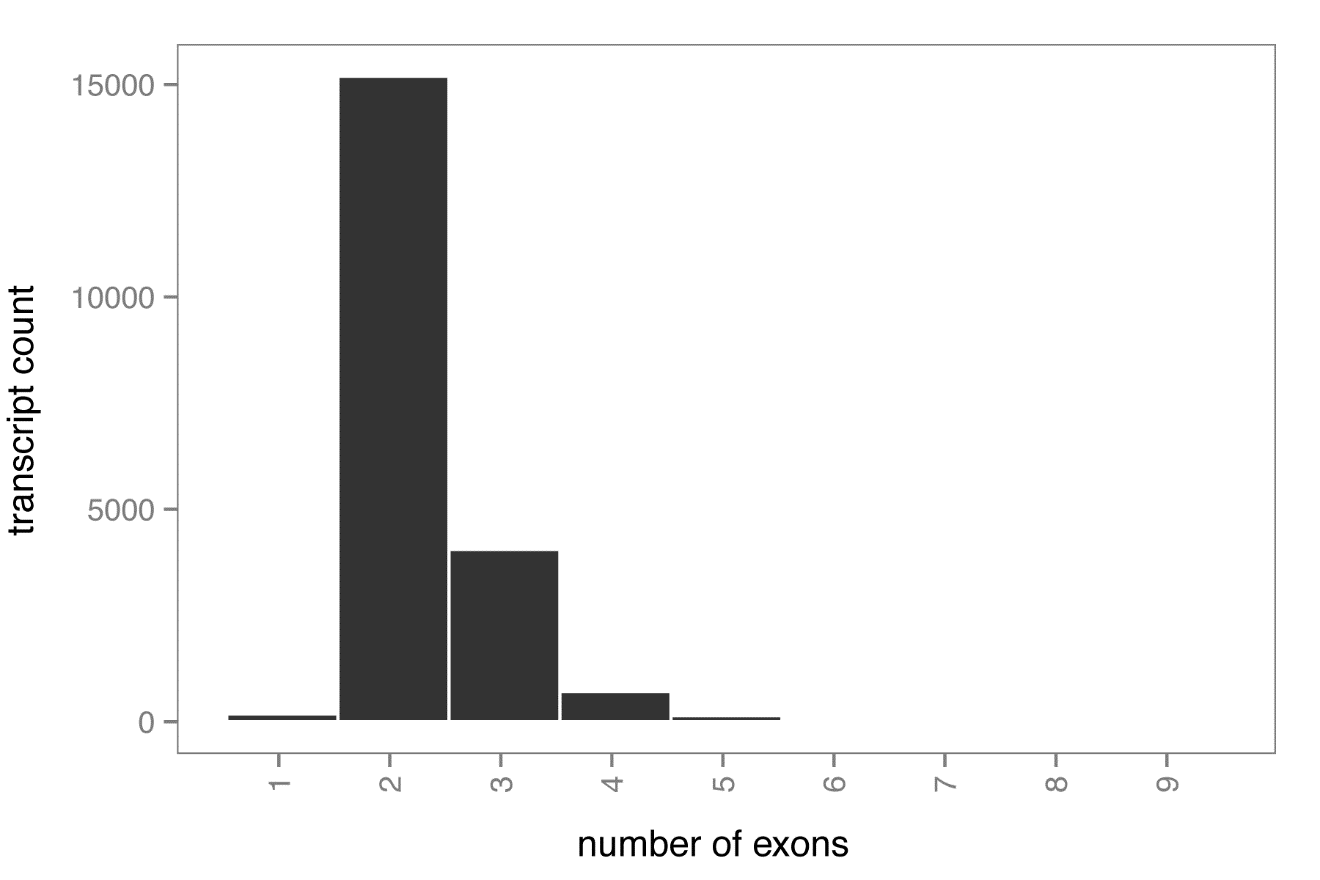


**Figure S2:** Distribution of the number of exons for the lncRNA transcripts in our dataset


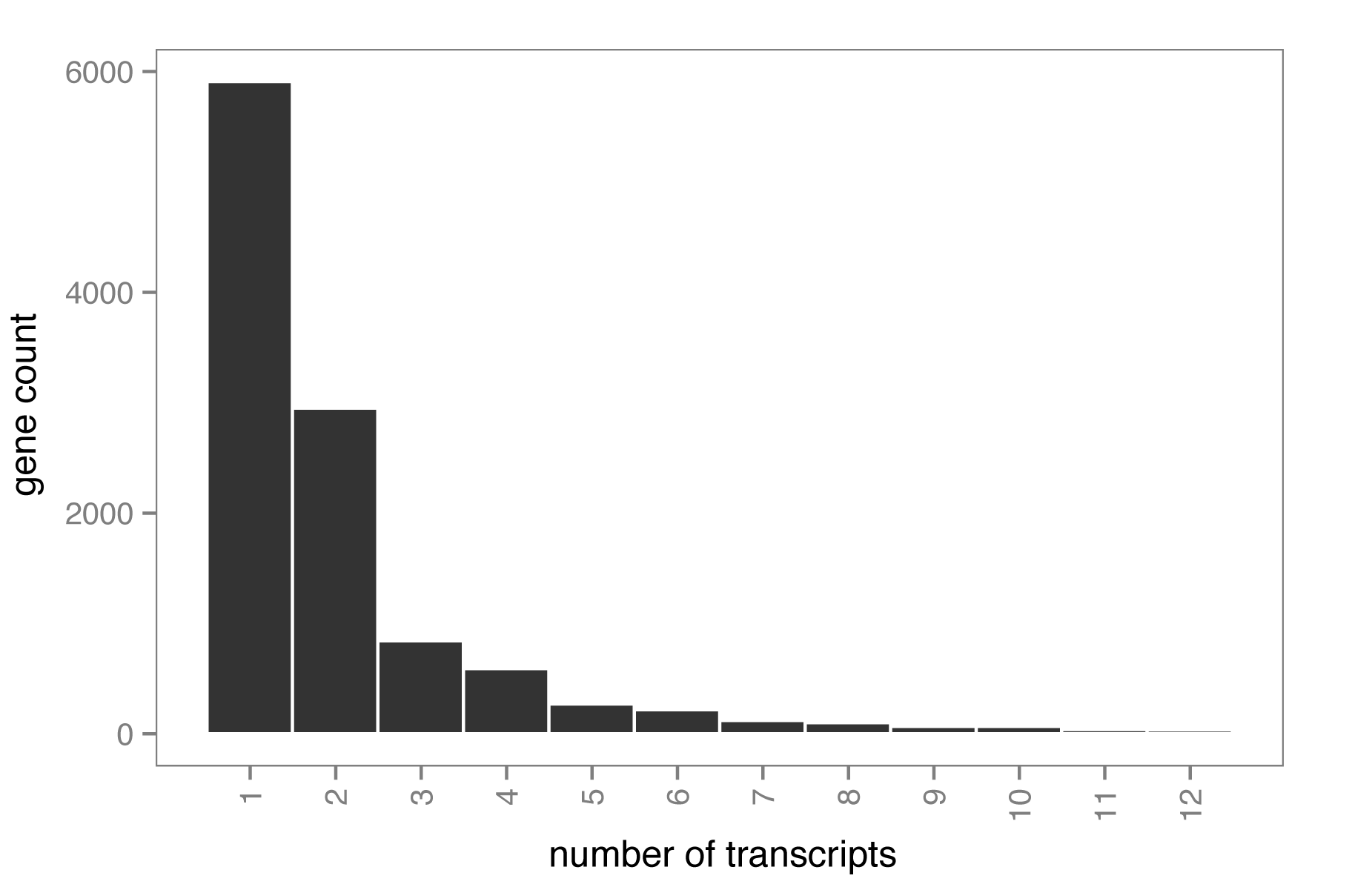


**Figure S3:** Distribution of the number of transcripts per lncRNA gene (locus)


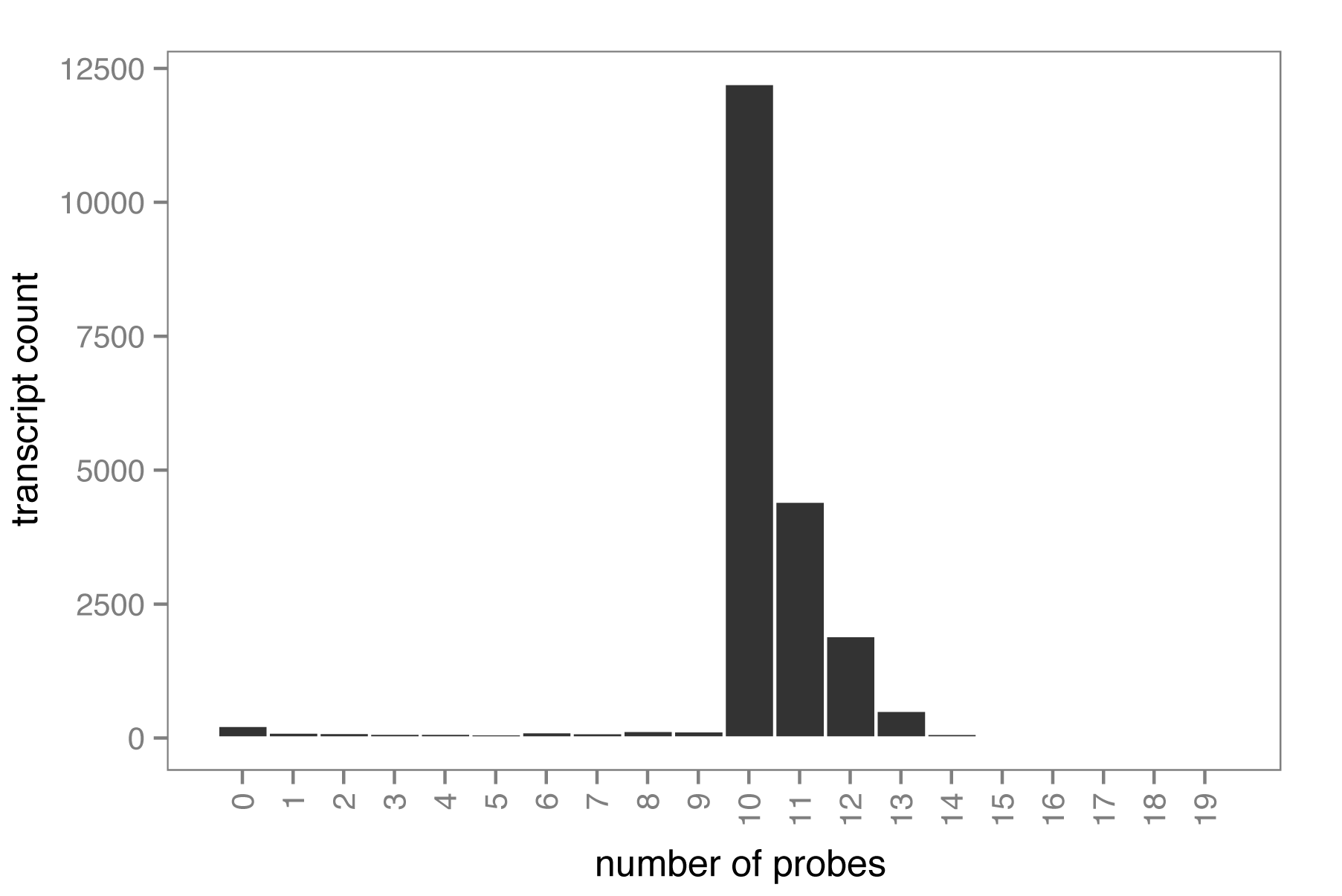


**Figure S4:** The required number of probes for a transcript depends on the number of exons. For transcripts with five exons or less, the required number of probes is 10. For larger transcripts, the required number is two times the number of exons. Only for a small fraction of transcripts, the pipeline failed to design at least 10 probes


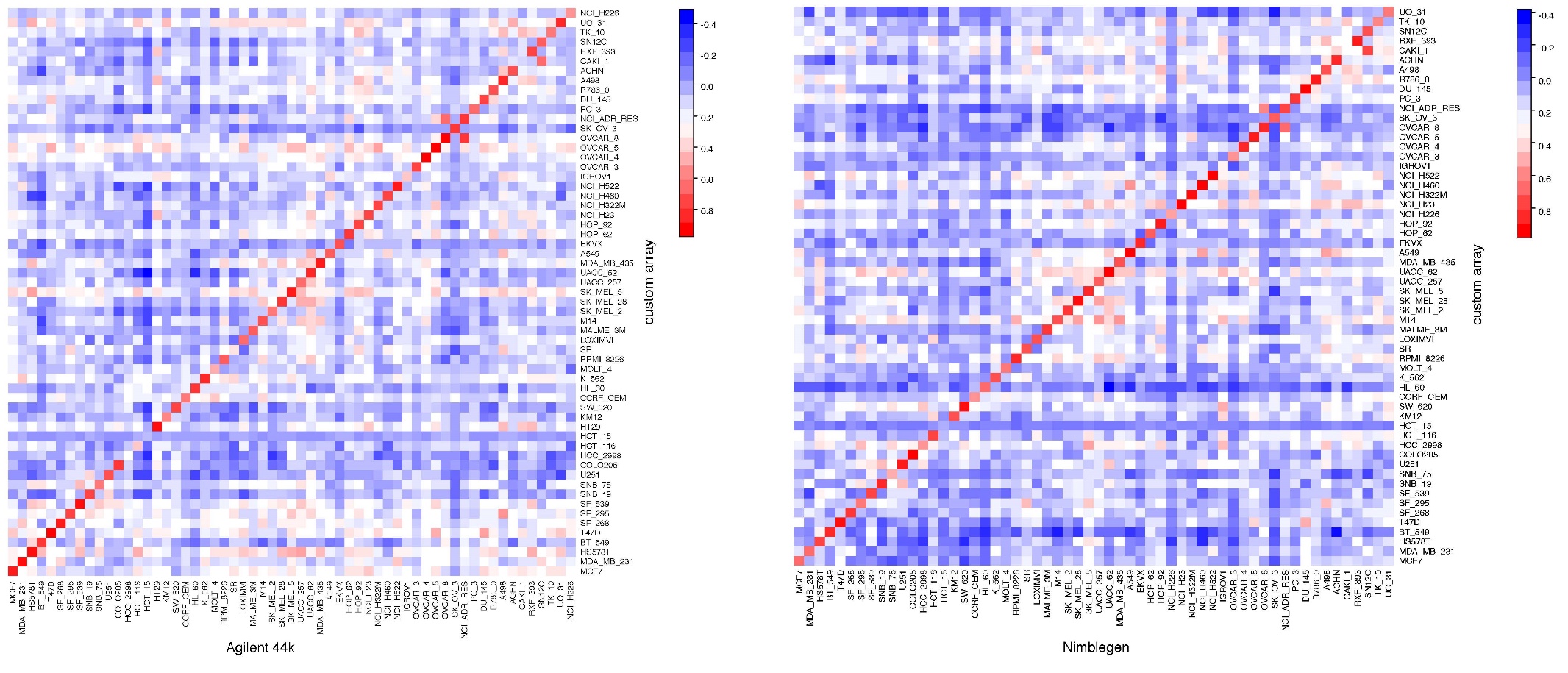


**Figure S5:** Comparison of the global copy number profiles (averaged in 1 Mb bins) with publically available profiles of two different array CGH platforms (Agilent 44K and Nimblegen 385k). Pearson correlation of all samples is depicted. Blue corresponds to no correlation while red is a high correlation. Excellent correlation if observed for our platform. As expected, cell lines derived from the same individual (such as NCI/ADR-RES and OVCAR-8) are also highly correlated. In addition, this analysis revealed problems with 2 DNA samples (HCT-15 and CAKI-1) that were unresolved by repeating the hybridization


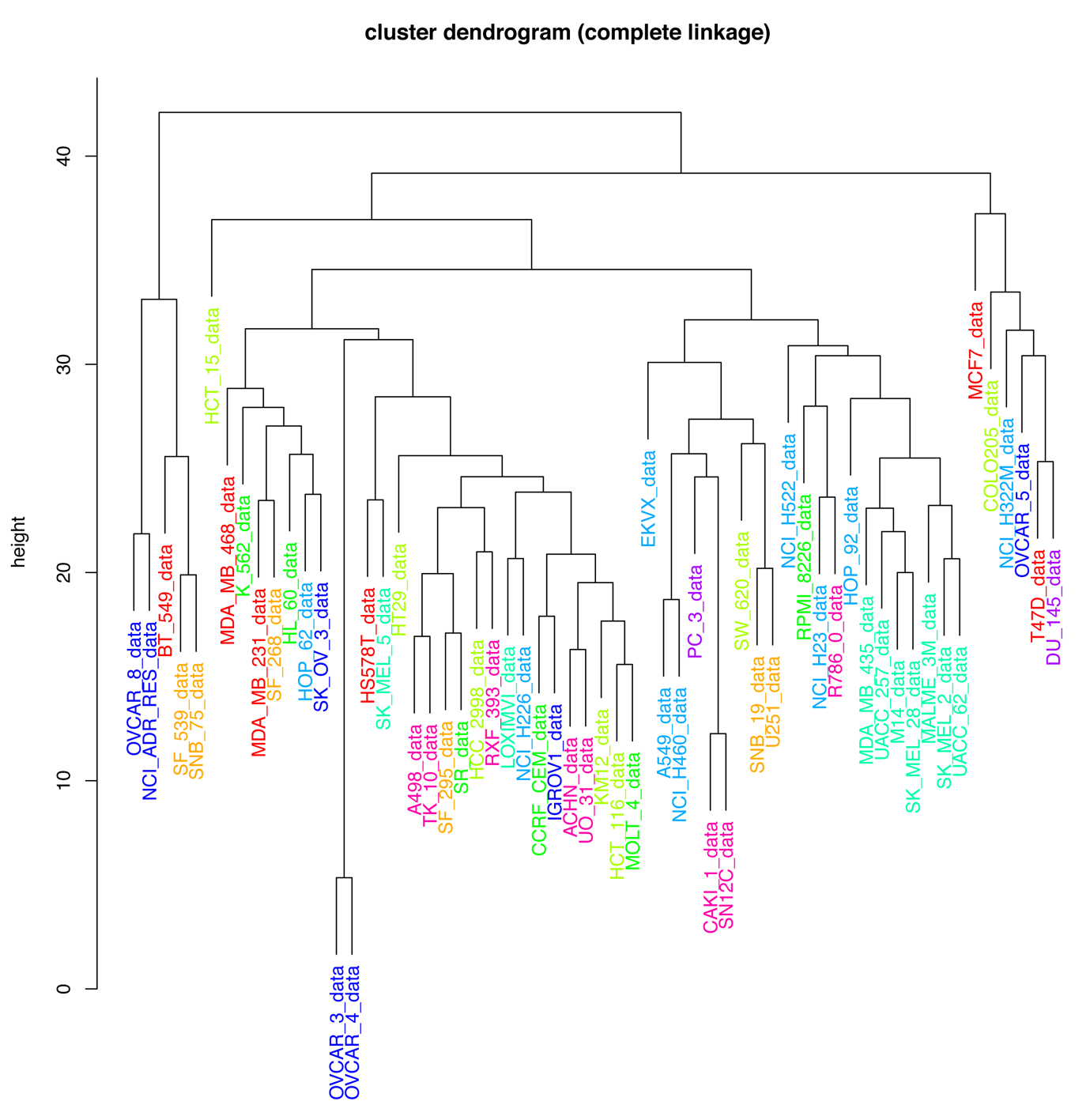


**Figure S6:** Complete linkage tree of the cell line based on their global copy number profiles. Cell lines derived from the same individual cluster closely together. On a cancer type level, only melanoma samples (green) cluster together


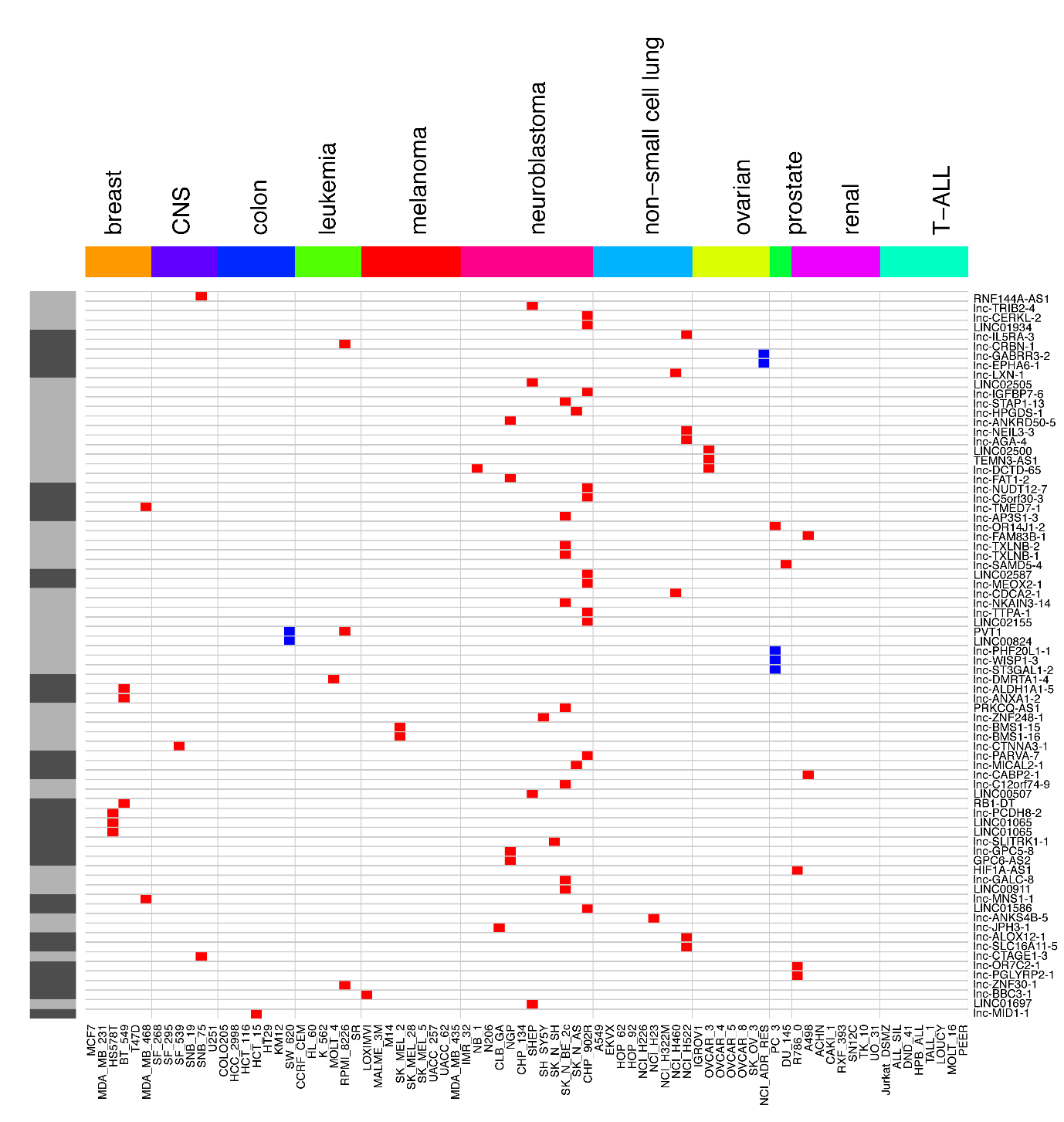


**Figure S7:** Overview of the lncRNA genes affected by focal SCNAs and copy number normal flanking protein coding genes. Red represents copy number loss (log-ratio < 1.5) in that cell line while blue corresponds to copy number gain (log-ratio > 1.5)


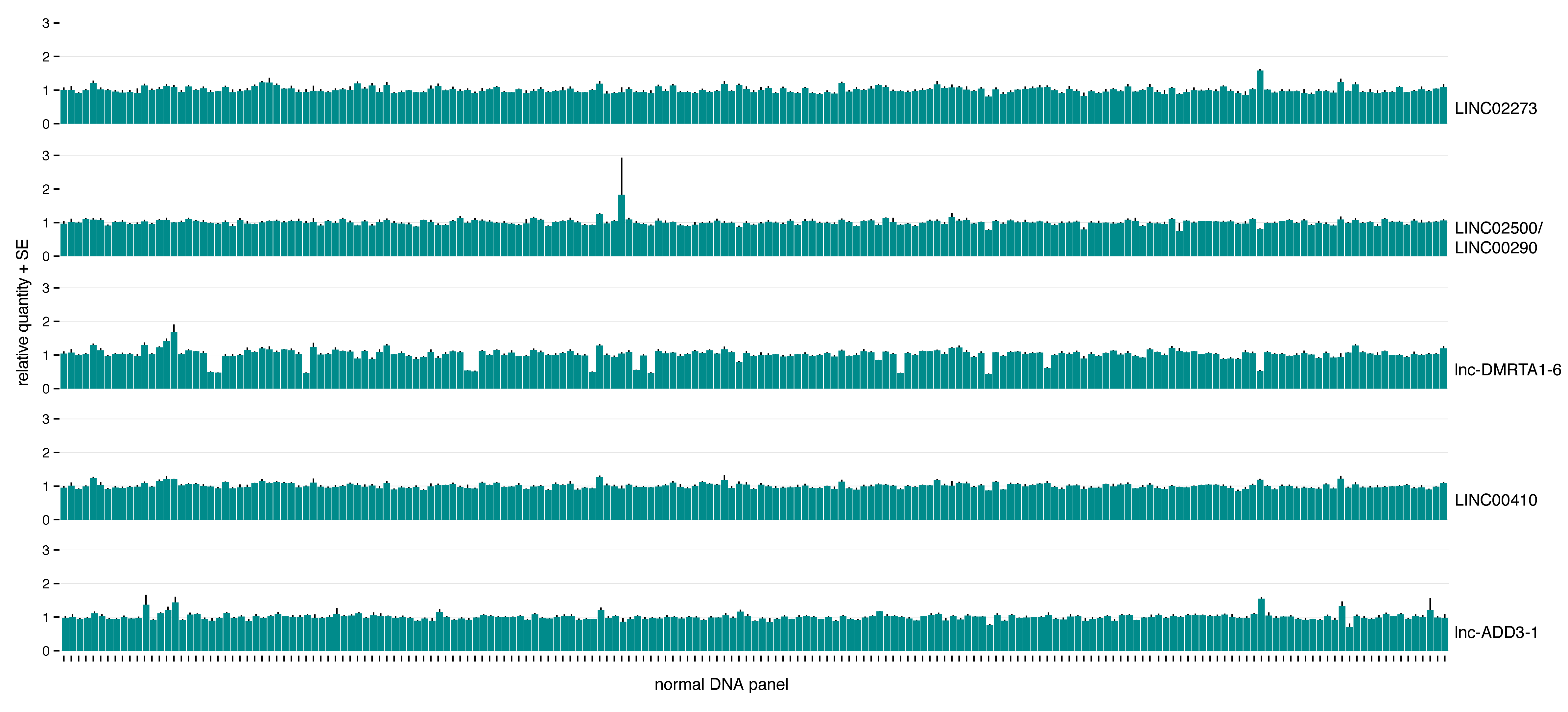


**Figure S8:** QPCR validation of 5 selected loci on 192 DNA samples from healthy individuals. Neither homozygous deletions nor high order amplifications could be detected for any lncRNA in any of the samples. However, heterozygous lnc-DMRTA1-6 deletions were present in 12 samples (6%).
